# Hardness–Deformation Energy Relationship in Metals and Alloys: A Comparative Evaluation Based on Nanoindentation Testing and Thermodynamic Consideration

**DOI:** 10.3390/ma14237217

**Published:** 2021-11-26

**Authors:** Masayuki Yamamoto, Masaki Tanaka, Osamu Furukimi

**Affiliations:** 1 Yamamoto Scientific Tool Lab. Co., Ltd., Funabashi, Chiba 273-0018, Japan; m.yamamoto@ystl.jp; 2 Department of Materials, Kyushu University, Nishi-ku, Fukuoka 819-0395, Japan; mukouile4309@gmail.com; 3 Center for Elements Strategy Initiative for Structural Materials, Kyoto University, Sakyo-ku, Kyoto 606-8501, Japan

**Keywords:** nanoindentation, hardness, elastic deformation energy, plastic deformation energy, elastic strain resistance

## Abstract

Nanoindentation testing using a Berkovich indenter was conducted to explore the relationships among indentation hardness (*H*), elastic work energy (*W*_e_), plastic work energy (*W*_p_), and total energy (*W*_t_ = *W*_e_ + *W*_p_) for deformation among a wide range of pure metal and alloy samples with different hardness, including iron, steel, austenitic stainless steel (*H* ≈ 2600–9000 MPa), high purity copper, single-crystal tungsten, and 55Ni–45Ti (mass%) alloy. Similar to previous studies, *W*_e_/*W*_t_ and *W*_p_/*W*_t_ showed positive and negative linear relationships with elastic strain resistance (*H*/*E*_r_), respectively, where *E*_r_ is the reduced Young’s modulus obtained by using the nanoindentation. It is typically considered that *W*_p_ has no relationship with *W*_e_; however, we found that *W*_p_/*W*_e_ correlated well with *H*/*E*_r_ for all the studied materials. With increasing *H*/*E*_r_, the curve converged toward *W*_p_/*W*_e_ = 1, because the Gibbs free energy should not become negative when indents remain after the indentation. Moreover, *H*/*E*_r_ must be less than or equal to 0.08. Thermodynamic analyses emphasized the physical meaning of hardness obtained by nanoindentation; that is, when *E*_r_ is identical, harder materials show smaller values of *W*_p_/*W*_e_ than those of softer ones during nanoindentation under the same applied load. This fundamental knowledge will be useful for identifying and developing metallic materials with an adequate balance of elastic and plastic energies depending on the application (such as construction or medical equipment).

## 1. Introduction

The mechanical properties of a material, particularly its strength and ductility, are the most fundamental metrics for evaluating its deformation behavior under applied stress. Ductility involves both uniform and local deformation, which occur by different mechanisms. Understanding the balance of these deformation modes is important for determining suitable materials for a given application. Hu et al. [1] showed that an increase in the local elongation of dual phase (DP) 980 steel correlated with an improvement in the hole expansion ratio obtained during hole piercing tests, classified as the edge stretchability. Taylor et al. [2] reported that the hole expansion ratio of DP980 steel decreased as the martensite hardness and martensite/ferrite hardness ratio (as determined by nanoindentation tests) increased. Furthermore, using synchrotron X-ray laminography of nanoscale precipitated steel and bainitic steel, Mugita et al. [3] showed that an increase in the nanoindentation hardness at the grain boundaries correlated with a decrease in local elongation, because the growth of voids was accelerated.

It is possible to analyze mechanical properties such as strength and elongation thermodynamically by integrating the stress–strain curve to determine the deformation energy [4,5]. However, thermodynamic analyses have rarely been used as a means of controlling the microstructure. Our research group has previously shown that the local deformation energy can be increased by controlling the microstructure of an alloy. Specifically, we clarified the effects of chromium-based steels with precipitations [6] and the austenite stability of duplex stainless steels [7] on the local deformation energy, as determined from the maximum load to fracture obtained from tensile stress–strain curves.

Hardness testing is widely used to evaluate the plastic deformability of materials. In the last few decades, nanoindentation testing methods [8,9,10,11,12,13,14,15,16,17,18] have been developed and are rapidly becoming widespread for the evaluation of various materials, including steels [19], tungsten [20], copper [21], and NiTi alloys [22]. This has produced new metallurgical knowledge about nanoscale deformation that is useful for the development of superior materials. Nanoindentation tests produce load–displacement curves that provide detailed information about the plastic deformation behavior during compression and unloading with nanoscale granularity, even under low loads (on the order of millinewtons). During the early development of nanoindentation testing techniques, Oliver and Pharr [23,24] proposed a method for determining the elastic modulus from the load–displacement curves. Sudden drops in deformation resistance during compression, called “pop-in events,” are sometimes observed in the load–displacement curves of nanoindentation tests [25]. This phenomenon is closely related to the dislocation nucleation behavior and indicates the transition point from elastic to elasto-plastic deformation [26]. Thus, nanoindentation testing can be applied in studies of both elastic and plastic deformation. Further, the elastic work energy (*W*_e_), plastic work energy (*W*_p_), and total work energy (*W*_t_ = *W*_e_ + *W*_p_) can be determined by integrating the load–displacement curves, as described in an ISO standard [27], and most testing devices can now automatically calculate these energy values. Analyzing the deformation energy from hardness test results is therefore a valuable way of determining the relationships between the mechanical properties and microstructure of a material based on the thermodynamic properties.

Okoro et al. [28] added multi-walled carbon nanotubes (MWCNTs) to sintered Ti6Al4V-based nanocomposites and investigated the effect on the nanoindentation hardness (*H*), plasticity index (*W*_p_/*W*_t_), recovery index (*W*_e_/*W*_t_), and elastic strain resistance (*H*/*E*_r_). *E*_r_ is the Young’s modulus obtained by nanoindentation (*E*_r_ = π^1/2^/2 × *S*/*A*_c_^1/2^, where *S* is the slope of the force–displacement curve during unloading, and *A*_c_ is the projected area of the indent). Their findings revealed that the addition of MWCNTs improved *H*, *W*_e_/*W*_t_, and *H*/*E*_r_, but decreased *W*_p_/*W*_t_. The anti-wear resistance was also improved. Cheng and Cheng [29] used finite element method (FEM) numerical analysis and experimental verification to show that, for various materials, *H*/*E*_r_ has a negative linear relationship with *W*_p_/*W*_t_. In addition, Yang et al. [30] demonstrated that *H*/*E*_r_ has a positive linear relationship with *W*_e_/*W*_t_ for 20 sets of materials. The deformation energy obtained from nanoindentation tests is therefore a useful parameter for evaluating the deformation behavior of various materials. Moreover, it can be used in the development of new materials.

*W*_e_ and *W*_p_ represent the work required for different atomistical phenomena. Herein, *W*_e_ is the work required to change the atomic distance elastically, whereas *W*_p_ is the work required to move or multiplicate lattice defects such as dislocations and vacancies. The linear relationships between *W*_e_/*W*_t_ and *H*/*E*_r_ and between *W*_p_/*W*_t_ and *H*/*E*_r_ [29,30] strongly suggest that *W*_p_/*W*_e_ can also be regarded as a function of *H*/*E*_r_. The correspondence between *W*_p_/*W*_e_ and *H*/*E*_r_ indicates that the mechanisms of elastic and plastic deformation are related. To explore this hypothesis, we measured *H*, *W*_e_, and *W*_p_ by nanoindentation testing for six types of iron and steel, including austenitic stainless steels, with *H* values measured by nanoindentation (defined as *H*_IT_ in ISO 14577 [31]) of approximately 2600–9000 MPa. In addition, high purity copper, single-crystal tungsten, and NiTi alloy were used as test materials. This paper analyzes the respective relationships between *H*, *W*_e_, and *W*_p_ and determines the *W*_p_/*W*_e_ ratio as a metric for identifying materials with an adequate balance of elastic and plastic energies for a given application, such as construction beams and guidewires for medical equipment. Finally, thermodynamic analysis was performed to elucidate the relationships among these properties.

## 2. Experimental Methods

### 2.1. Test Materials

A total of nine test materials was used, including six types of iron and steel: martensitic steel (Vickers hardness reference block, HMV700; Yamamoto Scientific Tool Lab. Co., Ltd., Funabashi, Japan; Commercial); bainitic steel (JFE Steel Co., Ltd., HQ: Tokyo, Japan; Laboratory); interstitial-free steel (JFE Steel Co., Ltd., HQ: Tokyo, Japan; Commercial); electrodeposited iron (MAIRON Grade SHP; Toho Zinc Co., Ltd., HQ: Tokyo, Japan; Laboratory); and two austenitic stainless steels (JIS SUS304 and JIS SUS316; Sanyo Special Steel Co., Ltd., HQ: Himeji, Japan; Commercial). The remaining test materials were high purity copper (Vickers hardness reference block, HMV40 JIS C1020P; Yamamoto Scientific Tool Lab. Co., Ltd., Funabashi, Japan; Commercial), single-crystal tungsten (MaTecK GmbH, HQ: Jülich, Germany; Commercial), and 55Ni–45Ti (mass%) alloy (prepared in a vacuum melting using a high frequency induction heating furnace with minimal contamination; heat-treated at 773 K for 3600 s in air followed by quenching; Daido Steel Co., Ltd. HQ: Nagoya, Japan; Laboratory) (hereafter referred to as NiTi alloy).

Table 1 lists the chemical compositions of the test alloys, as obtained by spark discharge emission spectroscopic analyses and wet chemical analyses. The electrodeposited iron, high purity copper, and single-crystal tungsten had purities of 99.98%, 99.99%, and 99.99%, respectively.

### 2.2. Nanoindentation Tests

The dimensions and preparation methods of the nanoindentation test specimens are shown in Table 2. Each specimen was wet-polished using emery paper, in sequential order from 320 to 2000 grit, and then mirror polished using a diamond abrasive and colloidal silica.

**Figure 1:**
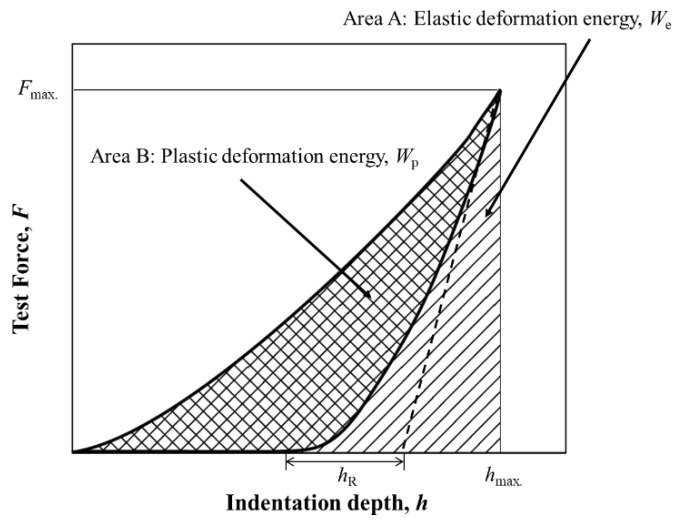
Definition of the elastic and plastic work energies based on load–displacement curves.

Nanoindentation tests were conducted using a nanoindentation hardness tester (Elionix Inc., ENT-2100) with a Berkovich indenter under a loading rate of 0.98 mN/s and maximum load of 9.8 mN. For each sample, *H*, *W*_e_, and *W*_p_ were determined from the load–displacement curve (see Figure 1): *H* was determined as the ratio of the maximum test load to the projected contact area of the indenter [31], and *W*_e_ and *W*_p_ were determined as the areas of regions A and B, respectively [27]. These values were calculated automatically by software included with the nanoindentation tester. These measurement methods are described in ISO 14577 [27]. The number of measurement points was set at 30 points per specimen, and the values shown in the tables and figures are averages of these 30 points. We excluded abnormal data such as those of indentions on grain boundaries.

## 3. Results

**Figure 2:**
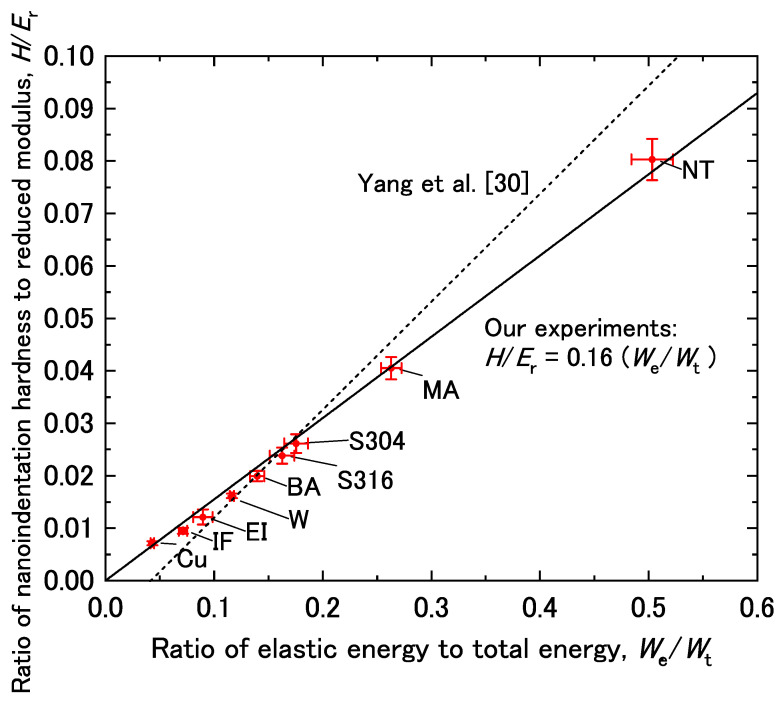
Ratio of nanoindentation hardness to reduced modulus (*H*/*E*_r_) as a function of the recovery index (*W*_e_/*W*_t_). The error bars are drawn with the standard deviation.

Table 3 shows the nanoindentation test results measured under a load of 9.8 mN along with yield strength (YS), and tensile strength (TS) measured by tensile tests. The relationship between *H*/*E*_r_ and *W*_e_/*W*_t_ was determined based on these results (solid line in Figure 2). The relationship between *H*/*E*_r_ and *W*_e_/*W*_t_ was linear and intercepted the origin. The linear coefficient was obtained by using the least-squares method, including the origin, as denoted by the following equation:

*H*/*E*_r_ = *α*(*W*_e_/*W*_t_) (1)

where *α* is the slope of the line, which had a value of 0.16 in this study.

Yang et al. [30] previously investigated the relationship between *H*/*E*_r_ and *W*_e_/*W*_t_ by using FEM and experimental verification. They also found a good linear relationship between *H*/*E*_r_ and *W*_e_/*W*_t_, which is shown as a dashed line in Figure 2. The gradient of the relationship obtained by Yang et al. is slightly higher than that in this study. This is because the regression line in their work was drawn directly from experimental data. However, they noted that the line should intercept the origin.

**Figure 3:**
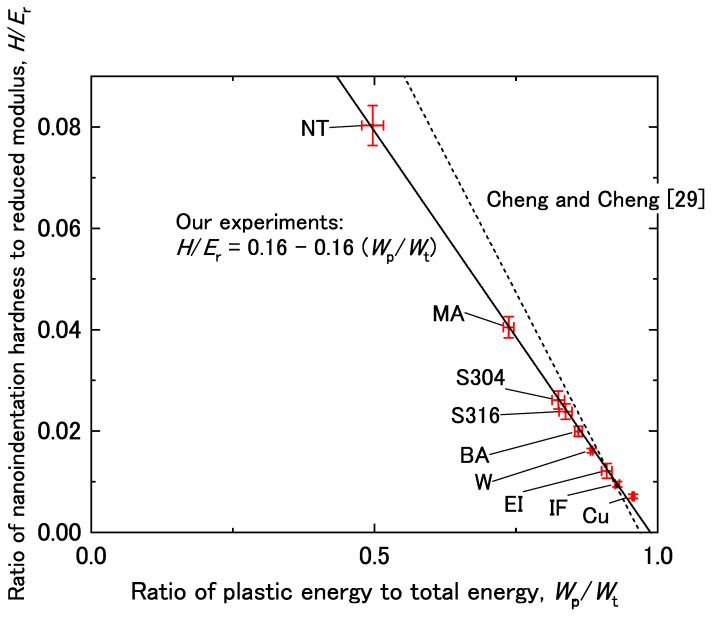
Ratio of nanoindentation hardness to reduced modulus (*H*/*E*_r_) as a function of the plasticity index (*W*_p_/*W*_t_). The error bars are drawn with the standard deviation.

Figure 3 shows the relationship between *H*/*E*_r_ and *W*_p_/*W*_t_ for the experimental data obtained in this study (solid line) and data obtained by Cheng and Cheng [29] (dashed line). The data obtained by Cheng and Cheng are based on FEM and experimental verification (nanoindentation tests using a conical-type indenter). Both sets of data identified a linear relationship between *H*/*E*_r_ and *W*_p_/*W*_t_. The linear relationship was approximated using the least-squares method as follows:

*H*/*E*_r_ = *β* + *γ* (*W*_p_/*W*_t_) (2)

where *β* and *γ* are constants, which had values of 0.16 and −0.16 in this study, respectively. Cheng and Cheng [29] also found a negative value of *γ* in Equation (2); however, the absolute value of *γ* obtained by Cheng and Cheng was slightly greater than that in this study.

**Figure 4:**
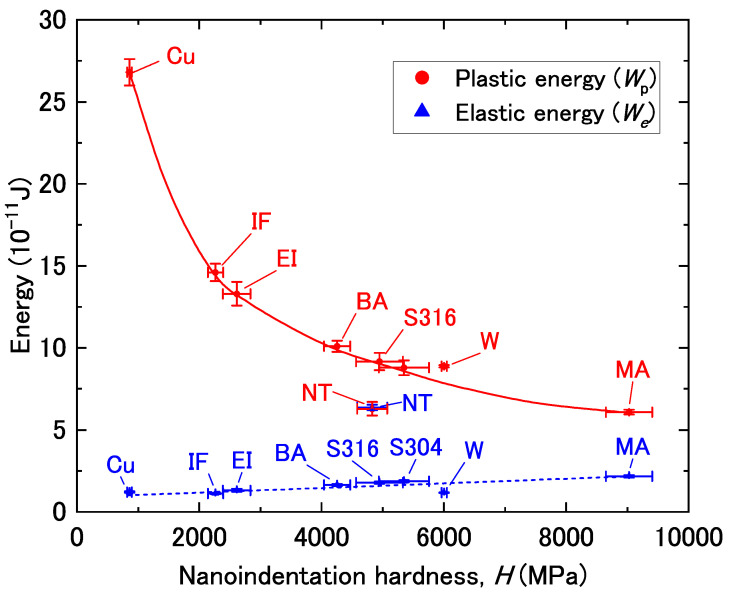
Elastic energy (*W*_e_) and plastic energy (*W*_p_) as a function of nanoindentation hardness (*H*). The error bars are drawn with the standard deviation.

As described above, *W*_e_/*W*_t_ and *W*_p_/*W*_t_ are both linearly related to *H*/*E*_r_, albeit in different directions (positive and negative). This suggests that there is a relationship between *W*_e_ and *W*_p_ with a basis in the mechanical properties. To investigate this further, we first plotted *W*_e_ and *W*_p_ against *H*, as shown in Figure 4. The *W*_e_ values were almost independent of *H* and similar for all tested materials, except for the NiTi alloy, which had a much larger *W*_e_ value than those of the other materials. The *W*_e_ value is determined from the Young’s modulus and elastic strain. Therefore, the larger *W*_e_ value for the NiTi alloy was attributed to the elastic strain contributing to a larger proportion of the same total strain because the Young’s modulus was lower. The Young’s moduli of NiTi, iron/steel (including stainless steel), copper, and tungsten are 60 GPa [32], 190–220 GPa [33], 105–130 GPa [33], and 370 GPa [33], respectively. Meanwhile, as shown in Figure 4, *W*_p_ decreased with increasing *H*, and the values for all materials followed a single curve (except the NiTi alloy, which showed a value below this curve).

**Figure 5:**
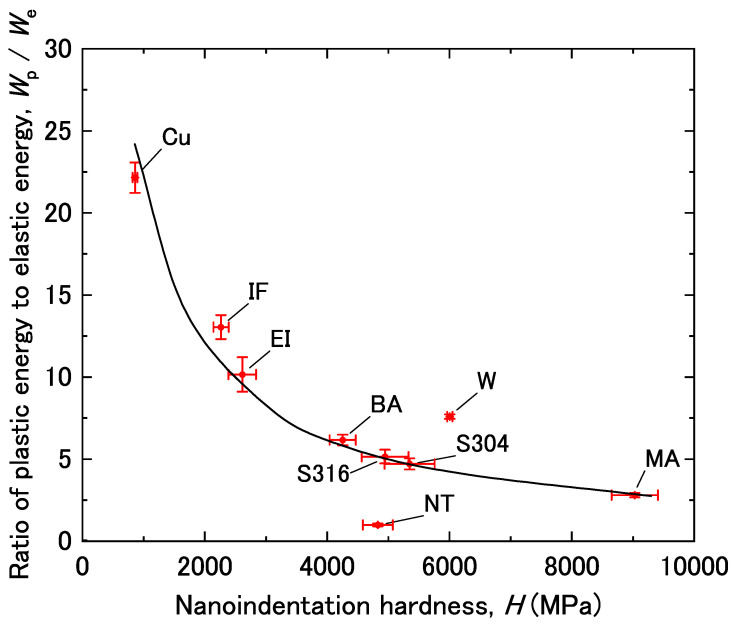
Ratio of plastic energy to elastic energy (*W*_p_/*W*_e_) as a function of nanoindentation hardness (*H*). The error bars are drawn with the standard deviation.

Because *H* is related to the yield strength, work-hardening coefficient, and plastic strain of a material, it is not always an effective parameter for evaluating *W*_e_. Therefore, we focused on the ratio of *W*_p_/*W*_e_ in order to account for the effect of plastic deformation. Figure 5 shows the relationship between *W*_p_/*W*_e_ and *H*. The results for iron, steel, and copper fell along a single curve, while the data for the NiTi alloy and tungsten deviated significantly from this curve.

The relationship between *H*/*E*_r_ and *W*_p_/*W*_e_ was determined by combining Equations (1) and (2) as follows:

(3) $$\frac{W_{p}}{W_{e}}=\left(1-\beta {\left(\frac{H}{E_{r}}\right)}^{-1}\right)\frac{\alpha }{\gamma }\;=-1+0.16{\left(\frac{H}{E_{r}}\right)}^{-1}$$

**Figure 6:**
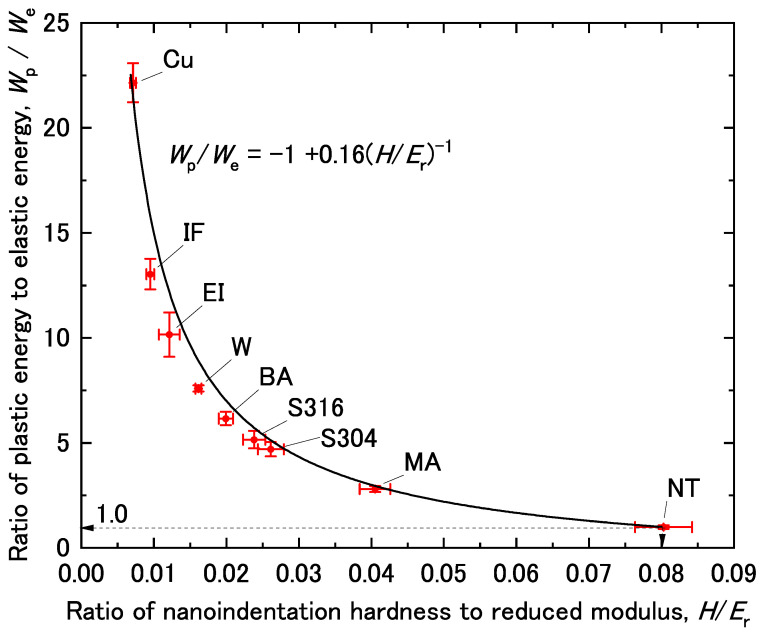
Ratio of plastic energy to elastic energy (*W*_p_/*W*_e_) as a function of the ratio of nanoindentation hardness to reduced modulus (*H*/*E*_r_). The error bars are drawn with the standard deviation.

According to this equation, *W*_p_/*W*_e_ can be represented as a function of *H*/*E*_r_. This relationship is plotted in Figure 6, along with the experimental data for all the materials used herein. Notably, all the materials used herein were accurately represented by Equation (3). Hence, *W*_p_/*W*_e_ was accurately determined from *H*/*E*_r_, where *W*_p_/*W*_e_ and *H*/*E*_r_ are both nondimensional values. It is to be noted that the maximum and minimum values of *H*/*E*_r_ and *W*_p_/*W*_e_ experimentally obtained in this sturdy were 0.08 and 1.0, respectively. The necessity of these values will be discussed next.

## 4. Discussion

This section considers the elastic and plastic deformation in the nanoindentation test from a thermodynamic perspective. Taking the specimen as the system, the change in Gibbs free energy (Δ*G*) due to an external force (i.e., indentation) is considered. Here, Δ*G* is generally given by the change in Helmholtz free energy (Δ*A*) and work (Δ*W*) as follows:

Δ*G* = Δ*A* + Δ*W* (4)

If the applied load in the nanoindentation test is low enough so that only elastic deformation occurs, no indent is left on the specimen surface after the test. In that case, the elastic work energy *W*_e_ is stored in the specimen as reversible work. Therefore, Δ*A* in Equation (4) corresponds to *W*_e_. Consequently, the change in Gibbs free energy for loading during elastic deformation, Δ*G*_elastic_, can be given as follows:

Δ*G*_elastic_ = Δ*A = W*_e_. (5)

If the applied load is large enough for plastic deformation to occur, an indent will be left on the surface after the test, i.e., the sample will undergo elasto-plastic deformation with a change in Gibbs free energy of Δ*G*_elasto-plastic_. Δ*A* is also given as *W*_e_, and Δ*W* corresponds to *W*_p_ in Equation (4). The sign of *W*_p_ is negative when the work done to the system is from an external force. Therefore,

Δ*G*_elasto-plastic_ = *W*_e_ − *W*_p_. (6)

Dividing both terms by *W*_e_ (≠0), Equation (6) can be given as

Δ*G*_elasto-plastic_/*W*_e_ = 1 − *W*_p_/*W*_e_. (7)

If *W*_p_/*W*_e_ < 1, Δ*G* is positive, and plastic deformation does not occur, because of an increase in the Gibbs free energy. In other words, only elastic deformation occurs during nanoindentation loading, and no indent would remain after unloading. It is therefore concluded that the condition *W*_p_/*W*_e_ ≥ 1 is required for an indent to be observed after the nanoindentation test. Consequently, Equation (8) gives the relationship between hardness and Young’s modulus:

*W*_p_/*W*_e_ = −1.0 + 0.16/(*H*/*E*_r_) ≥ 1

(8) $$∴\;H/E_{r}\;\leq \;0.08.$$

Figure 6 shows those conditions were well satisfied when indents were left on the surface after nanoindentation tests. Here, it should be noted that *W*_p_/*W*_e_ of the NiTi alloy used in this study was 0.99 with *H*/*E*_r_ ≈ 0.08, which is nearly the maximum value obtainable. It is also worth noting that Equation (8) reveals the physical meaning of hardness obtained by nanoindentation. That is, when *E_r_* is identical, harder materials show smaller values of *W*_p_/*W*_e_ than those of softer ones during nanoindentation under the same applied load.

It is expected that the relationship between *H*/*E*_r_ and *W*_p_/*W*_e_ should not be influenced by the microstructure, even though the microstructure has an important influence on each *H*/*E*_r_ and *W*_p_/*W*_e_. This is because the relationship between *H*/*E*_r_ and *W*_p_/*W*_e_ is universal, as shown in Figure 6, regardless of the specimen type. That is, even though the mechanical properties of a given material change with the microstructure, the *relationship* between *H*/*E*_r_ and *W*_p_/*W*_e_ should be maintained. The detail of the effects of microstructure will be explored further in our future work. In addition, the relationship might not be valid in alloys with special properties such as superplasticity or shape memory effect, such as that in NiTi alloys. Therefore, it will be interesting to investigate the effects of such special microstructures on the relationship between *H*/*E*_r_ and *W*_p_/*W*_e_.

The findings of this study may have widespread industrial applications. For example, it is known that an increase in *H*/*E*_r_ improves the wear resistance of a coating [34]. Based on the relationship between *H*/*E*_r_ and *W*_p_/*W*_e_, it is therefore reasonable that wear resistance will be improved when the value of *W*_p_ is decreased. In addition, it is to be stressed that Equation (8) indicates that there is an upper limit of *H*/*E*_r_ (or an upper limit of wear resistance) from a thermodynamic perspective.

## 5. Conclusions

Nanoindentation tests were performed to evaluate the relationships among the indentation hardness and energy components of various metals and alloys. The main conclusions are as follows.

(1) We/Wt and Wp/Wt are linearly correlated with H/Er with positive and negative relationships, respectively.
(2) For all the materials used in this study except NiTi alloy, We (Wp) increased (decreased) with increasing H.
(3) A good correlation between Wp/We and H/Er was found for all materials used in this study, where Wp/We and H/Er are nondimensional values. This emphasizes the physical meaning of hardness obtained by nanoindentation. That is, when Er is identical, harder materials show smaller values of Wp/We than those of softer ones during nanoindentation testing under the same applied load.
(4) Thermodynamic analyses showed that the indentation mark will be visible after nanoindentation testing when Wp/We ≥ 1, which suggests that the condition H/Er ≤ 0.08 should be true for any material.

## Figures and Tables

**Table 1:** Chemical composition of tested alloys (mass%).

Specimen	C	Si	Mn	Ni	Cr	Mo	Ti	Fe
Martensitic steel	0.86	0.16	0.25	0.01	0.04	-	-	bal.
Bainitic steel	0.09	0.7	1.5	-	-	-	0.12	bal.
Interstitial-free steel	0.002	0.002	0.14	-	-	-	0.046	bal.
Stainless steel (SUS304)	0.06	0.64	1.08	9.52	18.50	-	-	bal.
Stainless steel (SUS316)	0.06	0.56	1.36	12.34	17.57	2.4	-	bal.
NiTi alloy	-	-	-	55	-	-	45	-

**Table 2:** Dimensions (mm) and preparation of nanoindentation test specimens.

Specimen	Short Name	Preparation
Bainitic steel	BA	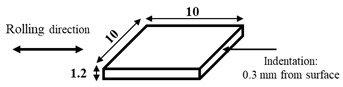
Interstitial-free steel	IF	
Stainless steel (SUS304)	S304	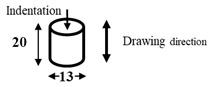
Stainless steel (SUS316)	S316	
Electrodeposited iron	EI	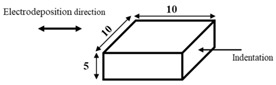
NiTi alloy	NT	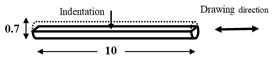
Single crystal tungsten	W	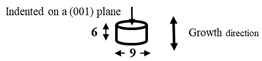
Martensitic steel	MA	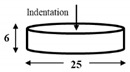
Copper	Cu	

**Table 3:** Average values of *H*, *W*_e_, *W*_p_, and *W*_p_/*W*_e_ obtained by nanoindentation tests (*F*_max_ = 9.8 mN; *n* = 30), along with yield stress (YS) and tensile strength (TS) obtained by tensile testes for various specimens.

Short Name	*H* (MPa)	*E*_r_ (GPa)	*W*_e_ × 10^−10^(J)	*W*_p_ × 10^−10^(J)	*W*_p_/*W*_e_	*YS* (MPa)	*TS* (MPa)
MA ^a^	9030 ± 379	223 ± 5.47	2.17 ± 0.08	6.08 ± 0.15	2.80 ± 0.14	-	-
BA	4255 ± 214	213 ± 8.18	1.64 ± 0.06	10.1 ± 0.33	6.16 ± 0.32	696	765
IF	2266 ± 124	238 ± 9.93	1.12 ± 0.04	14.6 ± 0.52	13.04 ± 0.73	188	275
EI	2613 ± 227	215 ± 13.4	1.31 ± 0.09	13.3 ± 0.72	10.15 ± 1.05	-	-
S304	5348 ± 411	205 ± 7.55	1.87 ± 0.07	8.79 ± 0.45	4.70 ± 0.34	368	658
S316	4948 ± 384	206 ± 7.65	1.78 ± 0.07	9.17 ± 0.53	5.15 ± 0.42	329	635
W	6006 ± 43.0	372 ± 8.82	1.17 ± 0.02	8.88 ± 0.07	7.59 ± 0.14	-	-
Cu ^b^	860 ± 38.3	121 ± 8.09	1.21 ± 0.04	26.8 ± 0.81	22.15 ± 0.92	65	213
NT	4829 ± 247	60 ± 1.68	6.37 ± 0.16	6.30 ± 0.42	0.99 ± 0.08	-	1500

^a^ Vickers hardness reference block HMV700. ^b^ Vickers hardness reference block HMV40 annealed at 613 K for 7200 s (Grain diameter ≒ 50 μm).
